# Segmental and global myocardial work in hypertensive patients with different left ventricular geometry

**DOI:** 10.1186/s12947-023-00310-y

**Published:** 2023-06-27

**Authors:** Huimei Huang, Liyun Fu, Qinyun Ruan, Ziling You, Lei Yan

**Affiliations:** 1 Department of Ultrasound, the First Affiliated Hospital of Fujian Medical University, No.20 Chazhong Road, Fuzhou, 350005 China; 2 Department of Ultrasound, National Regional Medical Center, Binhai Campus of the First Affiliated Hospital, Fujian Medical University, Fuzhou, 350212 China

**Keywords:** Hypertension, Geometry, Myocardial work, Myocardial function, Echocardiography

## Abstract

**Background:**

Myocardial work acquired by echocardiography has emerged as a novel method to evaluate myocardial function. We investigated global and segmental myocardial work in hypertension (HT) among patients with different patterns of left ventricular (LV) geometry in order to analyze the contribution of segmental myocardial work to global myocardial work.

**Methods and results:**

One hundred twenty-five patients with HT were divided into 4 groups: normal geometry (NG), concentric remodeling (CR), concentric hypertrophy (CH) and eccentric hypertrophy (EH). Longitudinal strain (LS) and the following indices were obtained by echocardiography: myocardial work index (MWI), myocardial constructive work (MCW), myocardial wasted work (MWW), and myocardial work efficiency (MWE). The global longitudinal strain (GLS) decreased gradually among the groups: NG, CR, CH and EH (*P* < 0.001). Global MWI (GWI) and global MCW (GCW) did not change across the different LV remodeling groups. Global MWW (GWW) increased and global MWE (GWE) decreased in both CH and EH group (*P* < 0.001). The LS of basal and middle regions reduced gradually in all HT subgroups, while apical LS decreased only in the CH and EH groups (*P* < 0.001). Basal MWI and MCW decreased in the CH and EH groups (*P* = 0.025, 0.007, respectively). Apical MWI and MCW increased in the NG and CR groups (*P* = 0.015, 0.044, respectively), with a decreasing trend in the CH and EH groups. All segmental MWW elevated and MWE reduced significantly in the CH and EH groups (*P* < 0.001). Univariate and multivariate logistic regression analyses demonstrated a significant association between left atrial volume index (LAVI), GLS, GWE and LV hypertrophy. At the receiver operating characteristic (ROC) analysis, optimal cutoff values of GLS, Apical LS, GWE and Apical MWE discriminating LV hypertrophy were 0.9072, 0.8049, 0.8325 and 0.7414, respectively.

**Conclusion:**

Apical myocardial work increases in the early stages of LV remodeling, likely as a compensatory mechanism to maintain normal global myocardial work. Segmental myocardial work analysis offers a reliable means to explore the distribution of myocardial impairment in hypertensive patients at different LV remodeling stages.

**Graphical Abstract:**

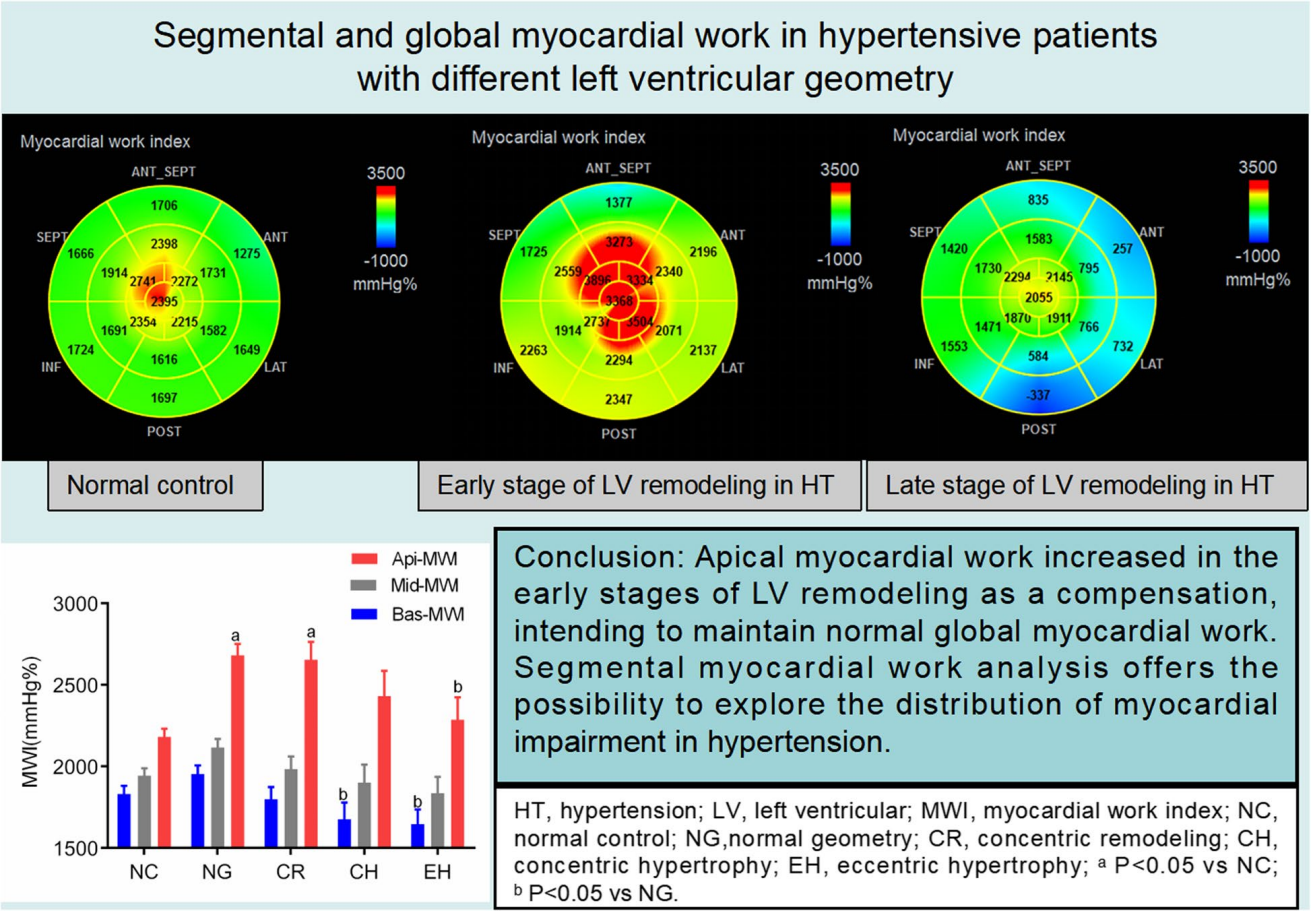

**Supplementary Information:**

The online version contains supplementary material available at 10.1186/s12947-023-00310-y.

## Introduction

Myocardial work acquired by 2-dimensional speckle tracking echocardiography (2D-STE) is a novel parameter to evaluate left ventricular (LV) myocardial performance. It takes into account the influence of afterload and myocardial deformation on LV systolic function [1–3]. Recent studies have revealed that [4, 5] the global myocardial work index (GWI) may identify coronary heart disease or other heart diseases with a high afterload more sensitively than global longitudinal strain (GLS). Parameters of myocardial work may be superior to strain in estimating changes in LV systolic function [2, 6, 7]. In hypertension (HT), both the elevated systolic blood pressure and ventricular remodeling can affect myocardial function [3]. The elevated systolic blood pressure may lead to the remodeling of LV structure and function [8]. How myocardial work is affected during LV remodeling in HT and how segmental myocardial work contributes to global myocardial work remains to be fully understood. This study aimed to observe the myocardial work of hypertensive patients with different LV geometries, using an LV pressure-strain loop (PSL) obtained by 2D-STE and non-invasive blood pressure measurement. This work also sought to analyze the contribution of segmental myocardial work to global myocardial work.

## Methods

### Study population

One hundred twenty-five essential hypertensive patients (aged 57.39 ± 10.93, 86 males and 39 females) who visited the First Affiliated Hospital of Fujian Medical University from June 2021 to March 2022 were enrolled in this retrospective study. The diagnosis of essential HT was designated according to 2018 ESC/ESH Guidelines for the management of arterial HT [9]. Normal controls (NC) included 42 healthy volunteers (age 55.62 ± 9.30, 19 males and 23 females) who were free of cardiovascular or systemic diseases. Patients were excluded for the following reasons: secondary HT, known coronary artery disease (history of ischemic heart disease or coronary stenosis > 50% at the coronary angiography examination), valvular stenosis and regurgitation, idiopathic cardiomyopathy, congenital heart disease, atrial fibrillation, or poor image quality.

The enrolled hypertensive patients were divided into 4 groups according to left ventricular mass index (LVMI) and relative wall thickness (RWT): Normal geometry (NG): normal LVMI and normal RWT, *n* = 36; Concentric remodeling (CR): normal LVMI and increased RWT, *n* = 20; Concentric hypertrophy (CH): increased LVMI and increased RWT, *n* = 24; Eccentric hypertrophy (EH): increased LVMI and normal RWT, *n* = 45. The classification of LV geometry and the calculation of LVMI and RWT were made according to 2015 recommendations for cardiac chamber quantification by echocardiography in adults [10], where LVMI > 115 (male) or > 95 g/m^2^ (female) was defined as hypertrophy. RWT was calculated using the formula (2 × posterior wall thickness) / (LV internal diameter at end-diastole), and RWT < 0.42 was defined as normal [10].

### Echocardiographic analysis

#### Conventional echocardiographic study

Comprehensive transthoracic echocardiography was performed using a GE Vivid E95 ultrasound scanner (GE Vingmed Ultrasound, Horten, Norway) equipped with a M5s probe (1.7–4.0 MHz). Frame rate was 50–80 frames per second. Patients were examined at rest and in the left lateral decubitus position. Electrocardiogram-triggered echocardiographic data were acquired and digitally stored in a cine-loop format for offline analysis. The following conventional parameters were measured in standard parasternal LV long axis views, apical 4-chamber, 2-chamber and 3 chamber views: left ventricular internal dimension in diastole (LVIDd), left ventricular internal dimension in systole (LVIDs), interventricular septal thickness (IVST), left ventricular posterior wall thickness (LVPWT), left atrial volume (LAV), left ventricular end diastolic volume (LVEDV), left ventricular end systolic volume (LVESV), peak mitral orifice flow velocity at early (E) and late (A) diastole, left ventricular ejection fraction (LVEF) calculated by biplane Simpson method, the average value of septal and lateral mitral annulus velocity at early diastole (e’), and the E/A and E/e’ ratios. LVM, LVEDV, LAV were indexed to body surface area (BSA). Echocardiographic examination was performed immediately after brachial cuff blood-pressure measurement, which was assumed to be equal to peak systolic LV pressure.

#### Longitudinal strain and myocardial work analysis

Image cine-loops were analyzed by a dedicated software (EchoPAC Software, version 203, General Electric Vingmed Ultrasound). In the automatic functional imaging mode, myocardial deformation measurements were performed using tissue speckle tracking and the displacement of speckles of myocardium in each spot was analyzed and tracked frame by frame. After manual tracing of the endocardial border of the end-systolic frame (determined at the aortic valve closure time) and adjusting the appropriate region of interest (ROI) (i.e. the width of the ROI was adjusted to fit the wall thickness as required) between the endocardium and epicardium, a bull's-eye map of the overall longitudinal strain (LS) of LV was obtained by automatic frame-by-frame tracking of the acoustic markers in the myocardial tissue. The ROI was adjusted manually until the tracking quality was scored as acceptable. After inserting the blood pressure measurements manually, the software then constructed a non-invasive LV PSL according to the duration of isovolumic and ejection phases defined by valvular timing events, which were defined by the opening and closure of the mitral and aortic valves. The area within the PSL provided an index of myocardial work as described by Russell et al. [11]. The following parameters were subsequently calculated [6]: GLS (LS values for all six LV myocardial segments in each of the apical 4-chamber, 2-chamber, and 3-chamber views, calculated by averaging values of the 18 segments), GWI, global myocardial constructive work (GCW), global myocardial wasted work (GWW), and global myocardial work efficiency (GWE). Myocardial work index (MWI) was defined as myocardial work within the area of the LV PSL calculated from mitral valve closure to mitral valve opening and myocardial constructive work (MCW) as work contributing to LV ejection. Myocardial wasted work (MWW) was defined as work that does not contribute to LV ejection. Myocardial work efficiency (MWE) was calculated as MCW/ (MCW + MWW).

To enable the investigation of regional strain and work distribution in the LV, the ventricle was divided into 18 segments. Three regions (the apical, middle, basal region) were each divided into six segments, enabling an investigation of the regional characteristics in each plane [3]. The measurement of each region was the average value of each of the 6 segments. The following parameters were subsequently calculated: longitudinal strain of basal segments, middle segments, apical segments (Bas-LS, Mid-LS, Api-LS); myocardial work index of basal segments, middle segments, apical segments (Bas-MWI, Mid-MWI, Api-MWI); myocardial constructive work of basal segments, middle segments, apical segments (Bas-MCW, Mid-MCW, Api-MCW); myocardial wasted work of basal segments, middle segments, apical segments (Bas-MWW, Mid-MWW, Api-MWW); myocardial work efficiency of basal segments, middle segments, and apical segments (Bas-MWE, Mid-MWE, Api-MWE). LS was analyzed by absolute value.

### Statistical analysis

SPSS version 24 (IBM Corporation, Armonk, NY) was deployed to perform the statistical operations. Continuous variables were summarized as mean values ± standard deviation (SD). Categorical variables were presented as numbers and percentages. Analysis of variance (ANOVA) was used to assess multiple comparisons among groups. Post-hoc comparisons were assessed with the Bonferroni correction. Differences between categorical variables were analyzed using the χ^2^ test. Univariate and multivariate logistic regression analyses were performed to identify independent factors for LV hypertrophy. Odds ratio (OR), 95% confidence interval (95% CI), and *P* value were reported as the results of logistic regression analysis. Receiver operating characteristic (ROC) analysis was performed to identify the optimal cutoff point of GLS, GWE, Api-LS and Api-MWE to discriminate LV hypertrophy. Intraclass correlation coefficients (ICC) were calculated for interobserver and intraobserver agreement in 15 randomly selected patients. Myocardial strain was re-measured to calculate myocardial work parameters from the same images by two independent observers who were blinded to all other patient data. The intraobserver reproducibility was achieved by the same observer at 1-week interval between the first and second measurements. Graphpad prism 6.0 was used for all data graphing. A value of *P* < 0 0.05 was considered statistically significant.

## Results

### Patient characteristics

Clinical characteristics of the five groups are presented in Table 1. There were no inter-group differences for age, sex, height, weight and resting heart rate. There were no inter-group differences for HT duration and anti-hypertensive medication among HT subgroups. Compared with control subjects, patients with HT showed higher systolic and diastolic blood pressure (*P* < 0.001).

**Table 1:** Clinical characteristics

	NC(*n* = 42)	NG(*n* = 36)	CR(*n* = 20)	CH(*n* = 24)	EH(*n* = 45)	*P*
Age (years)	55.62 ± 9.30	54.56 ± 10.64	57.55 ± 9.21	60.08 ± 13.39	58.24 ± 10.61	0.254
Male, n (%)	19(45.2%)	25(69.4%)	14(70.0%)	16(66.6%)	31(68.8%)	0.111
Height (cm)	163.64 ± 6.50	166.67 ± 7.70	165.80 ± 7.07	165.08 ± 9.78	164.31 ± 6.95	0.442
Weight (kg)	62.27 ± 6.33	66.64 ± 10.96	66.93 ± 9.67	64.21 ± 12.54	65.47 ± 11.78	0.345
BSA (m^2^)	1.64 ± 0.105	1.71 ± 0.174	1.72 ± 0.156	1.68 ± 0.205	1.69 ± 0.184	0.330
HT duration (years)	-	5.69 ± 3.54	7.46 ± 5.22	9.92 ± 7.70	5.28 ± 1.91	0.064
Anti-hypertensive medications, n (%)						
CCB	-	9(25.0%)	7(35.0%)	3(12.5%)	7(15.6%)	0.205
Beta-blocker	-	3(8.3%)	0(0)	1(4.2%)	2(4.4%)	0.567
ACE-I	-	1(2.8%)	0(0)	2(8.3%)	2(4.4%)	0.538
ARB	-	3(8.3%)	6(30.0%)	5(20.8%)	4(8.9%)	0.074
Diuretic	-	0(0)	0(0)	1(4.2%)	1(2.2%)	0.567
SBP (mm Hg)	116.26 ± 10.34	137.97 ± 14.92^a^(*P* < 0.001)	135.37 ± 13.80^a^(*P* < 0.001)	149.50 ± 21.38^a^(*P* < 0.001)	141.88 ± 21.89^a^(*P* < 0.001)	< 0.001
DBP(mm Hg)	73.81 ± 8.34	88.03 ± 12.04^a^(*P* < 0.001)	85.34 ± 8.69^a^(*P* = 0.001)	87.63 ± 14.07^a^(*P* < 0.001)	86.35 ± 15.18^a^(*P* < 0.001)	< 0.001
Heart rate (b.p.m)	69.60 ± 12.41	69.59 ± 10.89	71.80 ± 10.16	68.88 ± 12.27	72.31 ± 15.47	0.760

*NC* Normal control, *NG* Normal geometry, *CR* Concentric remodeling, *CH* Concentric hypertrophy, *EH* Eccentric hypertrophy, *BSA* Body surface area, *HT* Hypertension, *ACE-I* Angiotensin-converting enzyme inhibitor, *ARB* Angiotensin receptor blocker, *CCB* Calcium channel blocker, *SBP* Systolic blood pressure, *DBP* Diastolic blood pressure, *b.p.m* Beats per minute

^a^*P* < 0.05 versus control group

### Standard echocardiographic characteristics

In Table 2, echocardiographic characteristics were compared between patients with HT and control subjects. Increased LVIDd and LVEDVI were observed in CH and EH groups (*P* < 0.001). Compared with controls, LVEF was significantly decreased in CH and EH groups (*P* < 0.001), a phenomenon preserved in NG and CR groups. Diastolic function parameters revealed significantly reduced e’ and increased LAVI in HT subgroups (*P* < 0.001). Moreover, E/e’ was significantly elevated in CH and EH groups (*P* < 0.001).

**Table 2:** Conventional echocardiography parameters

	NC(*n* = 42)	NG(*n* = 36)	CR(*n* = 20)	CH(*n* = 24)	EH(*n* = 45)	*P*
LVIDd (cm)	4.72 ± 0.37	4.92 ± 0.38	4.47 ± 0.37^b^	5.00 ± 0.48^ac^	5.63 ± 0.67^abcd^	< 0.001
IVST (cm)	0.87 ± 0.13	0.95 ± 0.10^a^	1.07 ± 0.12^ab^	1.35 ± 0.28^abc^	1.12 ± 0.21^abd^	< 0.001
LVPWT (cm)	0.78 ± 0.09	0.86 ± 0.09^a^	1.01 ± 0.09^ab^	1.24 ± 0.18^abc^	0.94 ± 0.12^abd^	< 0.001
RWT	0.33 ± 0.04	0.35 ± 0.04	0.45 ± 0.03^ab^	0.50 ± 0.07^abc^	0.34 ± 0.06^ cd^	< 0.001
LVMI (g/m^2^)	78.86 ± 18.14	91.09 ± 13.18^a^	93.76 ± 14.16^a^	159.15 ± 44.23^abc^	138.15 ± 33.89^abc^	< 0.001
LVEDVI (ml/m^2^)	42.73 ± 8.11	46.72 ± 14.18	40.57 ± 11.18	58.85 ± 17.19^abc^	61.23 ± 17.68^abc^	< 0.001
LAVI (ml/m^2^)	22.86 ± 8.22	27.94 ± 6.28	27.56 ± 6.21	35.76 ± 8.77^abc^	35.10 ± 11.17^abc^	< 0.001
e’ (m/s)	0.10 ± 0.02	0.08 ± 0.02^a^	0.07 ± 0.01^a^	0.06 ± 0.02^ac^	0.06 ± 0.02^abc^	< 0.001
E/e’	7.66 ± 2.77	9.63 ± 3.24	10.54 ± 2.92	12.72 ± 4.93^a^	13.57 ± 8.14^ab^	< 0.001
LVEF (%)	66.11 ± 3.81	63.24 ± 6.61	63.78 ± 4.42	58.01 ± 8.72^a^	55.41 ± 13.81^abc^	< 0.001

*NC* Normal controls, *NG* Normal geometry, *CR* Concentric remodeling, *CH* Concentric hypertrophy, *EH* Eccentric hypertrophy, *LVIDd* Left ventricular internal dimension in diastole, *IVST* Interventricular septal thickness, *LVPWT* Left ventricular posterior wall thickness, *RWT* Relative wall thickness, *LVMI* Left ventricular mass index, *LVEDVI* Left ventricular end diastole volume index, *LAVI* Left atrial volume index, *E* Peak mitral orifice flow velocity at early diastole, *e’* Average velocities of mitral annulus at early diastole, *LVEF* Left ventricular ejection fraction

^a^*P* < 0.05 versus control group

^b^*P* < 0.05 versus NG group

^c^*P* < 0.05 versus CR group

^d^*P* < 0.05 versus CH group

### Global longitudinal strain and myocardial work parameters

The GLS showed a gradual decrease (for absolute values) in the HT sub-groups (*P* < 0.001). GWI and GCW did not change across the different LV remodeling groups. GWW was increased and GWE was reduced in both CH and EH group (*P* < 0.001), as shown in Table 3.

**Table 3:** Global longitudinal strain and myocardial work parameters

	NC(*n* = 42)	NG(*n* = 36)	CR(*n* = 20)	CH(*n* = 24)	EH(*n* = 45)	*P*
GLS (%)	-22.29 ± 1.94	-20.24 ± 2.35^a^	-19.00 ± 3.51^a^	-16.31 ± 4.69^ab^	-16.39 ± 5.58^ab^	< 0.001
GWI(mm Hg%)	2037.67 ± 310.24	2219.97 ± 330.31	2058.65 ± 372.86	2001.67 ± 528.51	1923.93 ± 695.10	0.108
GCW(mm Hg%)	2296.81 ± 343.01	2494.81 ± 370.27	2339.10 ± 396.09	2231.29 ± 526.25	2177.89 ± 761.47	0.099
GWW(mm Hg%)	48.24 ± 29.37	66.19 ± 32.88	83.45 ± 89.33	138.04 ± 94.42^ab^	126.33 ± 88.65^ab^	< 0.001
GWE (%)	97.24 ± 1.38	96.56 ± 1.58	95.70 ± 3.18	92.50 ± 5.00^ac^	91.73 ± 7.86^abc^	< 0.001

*NC* Normal control, *NG* Normal geometry, *CR* Concentric remodeling, *CH* Concentric hypertrophy, *EH* Eccentric hypertrophy, *GLS* Global longitudinal strain, *GWI* Global myocardial work index, *GCW* Global myocardial constructive work, *GWW* Global myocardial wasted work, *GWE* Global myocardial work efficiency

^a^*P* < 0.05 versus control group

^b^*P* < 0.05 versus NG group

^c^*P* < 0.05 versus CR group

### Segmental analysis of longitudinal strain and myocardial work parameters

**Fig. 1:**
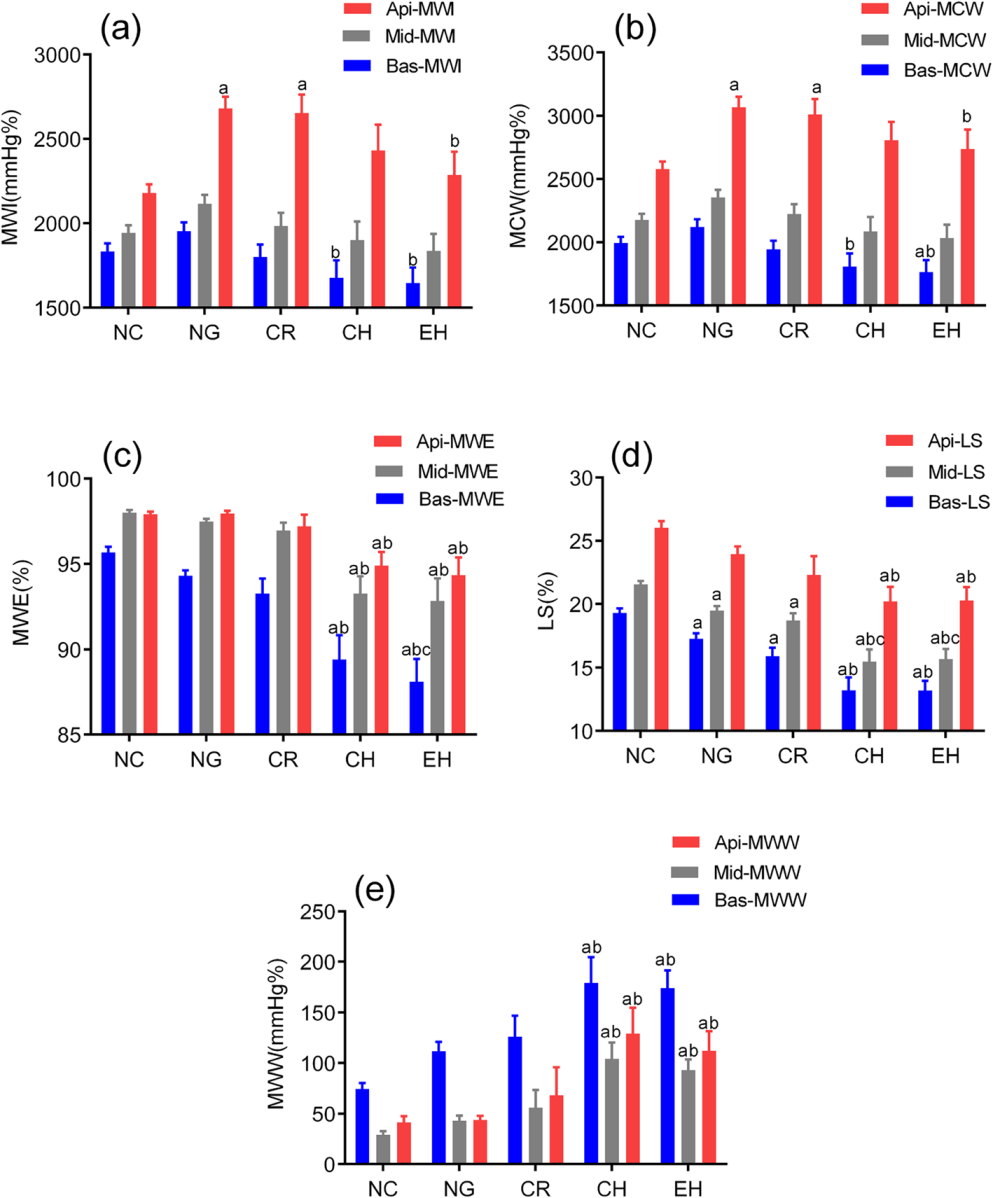
Segmental longitudinal strain and myocardial work parameters in HT and control subjects. Graph showed mean and error bars across all groups. **a** MWI, myocardial work index; **b** MCW, myocardial constructive work; **c** MWE, myocardial work efficiency; **d** LS, longitudinal strain (absolute value); **e** MWW, myocardial wasted work. ^a^ *P* < 0.05 vs control group, ^b^ *P* < 0.05 vs NG group, ^c^ *P* < 0.05 vs CR group; HT, hypertension; NC, normal control; NG, normal geometry; CR, concentric remodeling; CH, concentric hypertrophy; EH, eccentric hypertrophy; Bas-,basal segments; Mid-, middle segments; Api-, apical segments

**Fig. 2:**
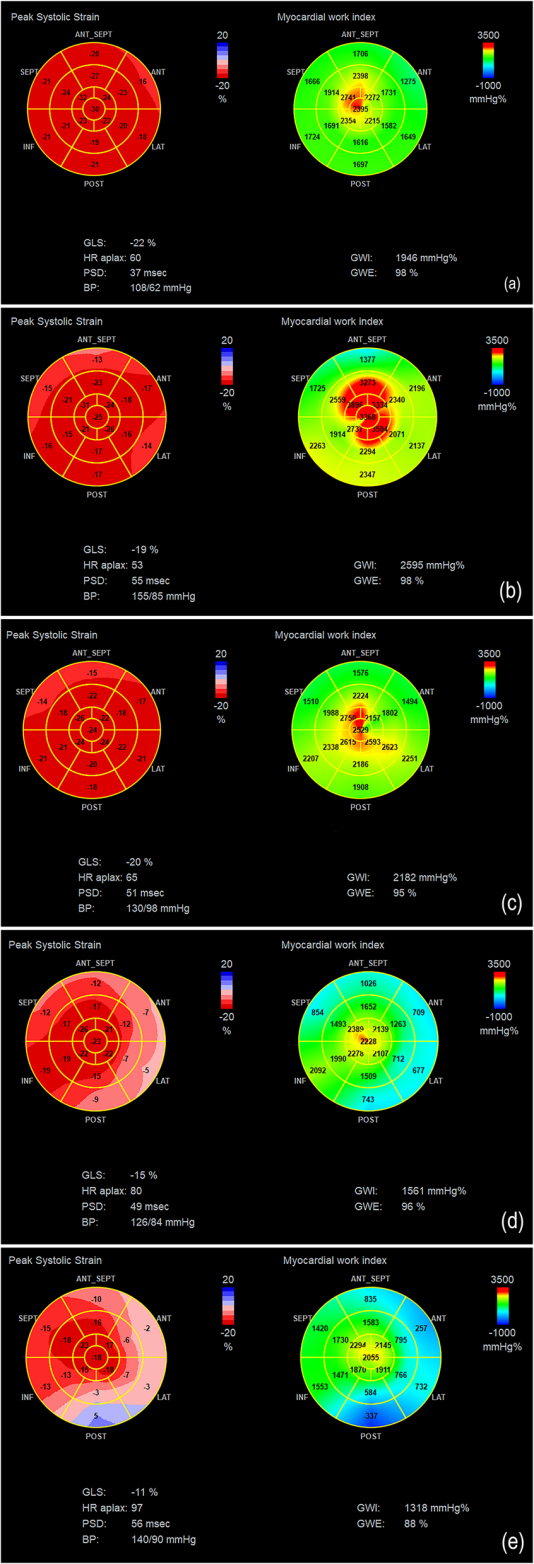
Left ventricular longitudinal strain and myocardial work. Examples of patients from: **a** Control group, **b** NG, normal geometry group, **c** CR, concentric remodeling group, **d** CH, concentric hypertrophy group, **e** EH, eccentric hypertrophy group. Each graph shows the bull's eye map of segmental longitudinal strain with low-moderate-high myocardial work coded in blue-pink-red, respectively (left); the bull's eye map of segmental myocardial work index with low-moderate-high myocardial work coded in blue-green–red, respectively (right); the parameters of global myocardial strain and myocardial work

Compared to controls, Bas-LS and Mid-LS were reduced gradually in all HT subgroups (*P* < 0.001), while Api-LS was decreased only in CH and EH groups. Basal MWI and MCW were decreased only in the CH and EH groups (*P* = 0.025, 0.007, respectively). Apical MWI and MCW were increased in the NG and CR groups (*P* = 0.015, 0.044, respectively). Middle MWI and MCW did not vary among the HT subgroups. All segmental MWW were elevated and all segmental MWE were reduced significantly in the CH and EH groups (*P* < 0.001) (Table 4, Figs. 1 and 2).

**Table 4:** Segmental analysis of longitudinal strain and myocardial work parameters

	NC(*n* = 42)	NG(*n* = 36)	CR(*n* = 20)	CH(*n* = 24)	EH(*n* = 45)	*P*
Bas-LS(%)	-19.30 ± 2.33	-17.30 ± 2.55^a^	-15.93 ± 2.98^a^	-13.21 ± 5.00^ab^	-13.19 ± 5.18^ab^	< 0.001
Mid-LS(%)	-21.54 ± 1.83	-19.47 ± 2.27^a^	-18.73 ± 2.43^a^	-15.49 ± 4.73^abc^	-15.70 ± 5.36^abc^	< 0.001
Api-LS(%)	-26.02 ± 3.23	-23.96 ± 3.57	-22.33 ± 6.51	-20.22 ± 5.60^ab^	-20.29 ± 7.04^ab^	< 0.001
Bas-MWI (mm Hg%)	1832.19 ± 311.46	1952.64 ± 318.29	1799.53 ± 335.11	1676.13 ± 503.18^b^	1646.14 ± 612.51^b^	0.025
Mid-MWI (mm Hg%)	1943.63 ± 285.17	2116.30 ± 321.85	1984.76 ± 346.22	1900.89 ± 537.25	1837.12 ± 671.35	0.117
Api-MWI (mm Hg%)	2180.88 ± 329.11	2681.93 ± 413.92^a^	2653.85 ± 487.32^a^	2431.44 ± 758.45	2286.50 ± 927.49^b^	0.015
Bas-MCW (mm Hg%)	1993.88 ± 310.36	2120.61 ± 368.17	1944.54 ± 308.78	1806.67 ± 506.48^b^	1763.94 ± 629.99^ab^	0.007
Mid-MCW (mm Hg%)	2176.67 ± 312.30	2356.17 ± 363.99	2222.49 ± 363.15	2086.64 ± 557.99	2032.16 ± 720.66	0.062
Api-MCW (mm Hg%)	2579.82 ± 374.04	3068.66 ± 499.24^a^	3010.60 ± 545.24^a^	2806.83 ± 714.15	2735.58 ± 1036.72^b^	0.044
Bas-MWW (mm Hg%)	74.53 ± 37.82	111.66 ± 55.69	125.89 ± 93.09	179.12 ± 125.10^ab^	174.08 ± 117.45^ab^	< 0.001
Mid-MWW (mm Hg%)	29.16 ± 22.89	43.10 ± 31.39	56.23 ± 78.86	104.40 ± 79.67^ab^	92.73 ± 70.83^ab^	< 0.001
Api-MWW (mm Hg%)	41.33 ± 40.26	43.56 ± 27.76	68.19 ± 123.58	129.08 ± 126.93^ab^	111.50 ± 131.63^ab^	< 0.001
Bas-MWE (%)	95.68 ± 2.10	94.25 ± 2.56	93.25 ± 4.37	89.45 ± 7.21^ab^	88.11 ± 9.54^abc^	< 0.001
Mid-MWE (%)	98.02 ± 1.34	97.47 ± 1.70	96.95 ± 2.88	93.26 ± 5.73^ab^	92.82 ± 9.74^ab^	< 0.001
Api-MWE (%)	97.90 ± 1.47	97.94 ± 1.10	97.21 ± 3.38	94.89 ± 4.07^ab^	94.34 ± 7.18^ab^	< 0.001

*NC* Normal control, *NG* Normal geometry, *CR* Concentric remodeling, *CH* Concentric hypertrophy, *EH* Eccentric hypertrophy, *Bas-* Basal segments, *Mid-* Middle segments, *Api-* Apical segments, *LS* Longitudinal strain, *MWI* Myocardial myocardial work index, *MCW* Myocardial myocardial constructive work, *MWW* Myocardial myocardial wasted work, *MWE* Myocardial myocardial work efficiency

^a^*P* < 0.05 versus control group

^b^*P* < 0.05 versus NG group

^c^*P* < 0.05 versus CR group

### Univariate and multivariate regression analyses

Uni- and multivariate logistic regression analyses showed significant association between LAVI, GLS, GWE and LV hypertrophy (Table 5). Higher LAVI, and lower GLS, GWE were associated with LV hypertrophy.

**Table 5:** Uni- and multivariable logistic regression analyses for LV hypertrophy

	Univariate regression analysis				Multivariate regression analysis			
	OR	95%CI		*P*	OR	95%CI		*P*
Age (years)	1.031	0.994	1.069	0.100	-	-	-	-
BSA (m^2^)	4.049	0.345	47.540	0.266	-	-	-	-
SBP (mm Hg)	1.114	1.067	1.163	< 0.001	1.124	0.983	1.285	0.088
LAVI (ml/m^2^)	1.177	1.104	1.254	< 0.001	1.193	1.052	1.354	0.006
LVEDVI (ml/m^2^)	1.109	1.062	1.158	< 0.001	1.084	0.972	1.208	0.147
e’(cm/s)	0.418	0.297	0.589	< 0.001	0.931	0.653	1.327	0.691
LVEF (%)	0.829	0.753	0.913	< 0.001	1.068	0.865	1.319	0.539
GLS (%)	1.963	1.482	2.600	< 0.001	3.173	1.436	7.011	0.004
GWE (%)	0.550	0.411	0.735	< 0.001	0.654	0.440	0.971	0.035
GWI (mm Hg%)	1.000	0.999	1.000	0.413	-	-	-	-
GCW (mm Hg%)	1.000	0.999	1.000	0.385	-	-	-	-
GWW(mmHg%)	1.031	1.017	1.046	< 0.001	1.000	0.871	1.147	0.996

*LV* Left ventricular, *OR* Odds ratio, *CI* Confidence interval, *BSA* Body surface area, *SBP* Systolic blood pressure, *LAVI* Left atrial volume index, *LVEDVI* Left ventricular end diastole volume index, *e’* average velocities of mitral annulus at early diastole, *LVEF* Left ventricular ejection fraction, *GLS* Global longitudinal strain, *GWE* Global myocardial work efficiency, *GWI* Global myocardial work index, *GCW* Global myocardial constructive work, *GWW* Global myocardial wasted work

### ROC analysis for hypertensive LV hypertrophy

**Fig. 3:**
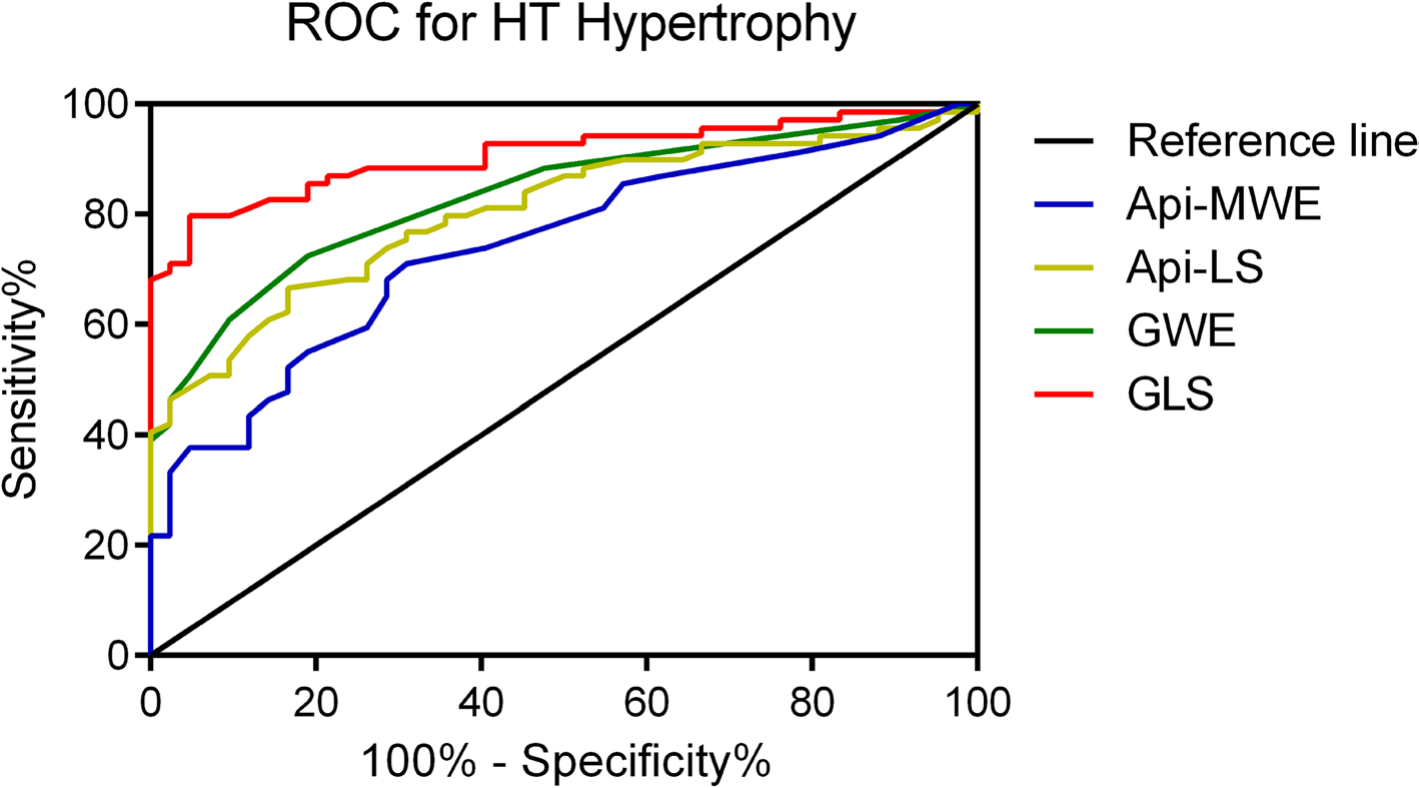
ROC analysis for hypertensive LV hypertrophy. Api-, apical segments; MWE, myocardial work efficiency; LS, longitudinal strain; GWE, global myocardial work efficiency; GLS: global longitudinal strain

The ROC curves of LS (GLS and Api-LS) and MWE (GWE and Api-MWE) were used for discriminating hypertensive LV hypertrophy, as shown in Fig. 3. The optimal cutoff point for GLS indicated LV hypertrophy was -19.92% [Area under the curve (AUC) 0.9072; 95%CI 0.8520 to 0.9624; sensitivity 79.71%; specificity 95.24%, *P* < 0.0001]. The optimal cutoff for GWE was 96.50% (AUC 0.8325; 95%CI 0.7585 to 0.9065; sensitivity 72.46%; specificity 80.95%, *P* < 0.0001). The optimal cutoff for Api-LS was -23.08% (AUC 0.8049; 95%CI 0.7253 to 0.8845; sensitivity 66.67%; specificity 83.33%, *P* < 0.0001). The optimal cutoff for Api-MWE was 97.92% (AUC 0.7414; 95%CI 0.6501 to 0.8326; sensitivity 71.01%; specificity 69.05%, *P* < 0.0001).

### Intra- and inter-observer reproducibility assessment

Fifteen patients were randomly selected, and the GWI, GCW, GWW were evaluated for intra- and inter-observer reproducibility. For GWI, the intra-observer ICC was 0.950 (95%CI 0.869–0.984, *P* < 0.001) and the inter-observer ICC was 0.950 (95%CI 0.858–0.983, *P* < 0.001). For GCW, the intra-observer ICC was 0.923 (95%CI 0.787–0.974, *P* < 0.001) and the inter-observer ICC was 0.938 (95%CI 0.827–0.979, *P* < 0.001). For GWW, the intra-observer ICC was 0.928 (95%CI 0.799–0.975, *P* < 0.001) and the inter-observer ICC was 0.929 (95%CI 0.802–0.975, *P* < 0.001).

## Discussion

Segmental myocardial work parameters can be measured by echocardiography [7], including MWI, MCW, MWW and MWE [6, 12, 13]. This study aimed to observe the myocardial work of hypertensive patients with different LV geometries, in order to analyze the contribution of segmental myocardial work to global myocardial work. The primary findings of our study were: (1) GLS decreased slightly during the early stages of LV remodeling (NG and CR groups), further deteriorating in the later stages (CH and EH groups). GWI and GCW did not change across the different LV remodeling groups. GWW increased and GWE decreased in the later stages of LV remodeling. (2) For segmental myocardial work: a) apical MWI and MCW increased during the early stages of LV remodeling, while basal and middle region MWI and MCW did not; at the late stages, apical MWI and MCW reduced to initial values, basal MWI and MCW decreased, while middle region MWI did not vary. b) For all the 3 myocardial regions, segmental MWW increased and MWE decreased at the late LV remodeling stages.

### Influences of HT on myocardial work and the possible mechanism

In accordance with previous studies [14, 15], we found that the severity of LV remodeling was associated with poor diastolic function. Elevated myocardial work was observed in hypertensive patients with preserved LVEF and GLS [16]. With mild to severe HT, GWW increased and GWE decreased gradually [6, 16]. HT has an important influence not only on myocardial work, but also LV remodeling. LV end-systolic stiffness is an index of myocardial contractility, which reflects the ability of the LV to pump against a higher pressure associated with enhanced myocardial contractility [17]. The increased GWI allows the preservation of LVEF and GLS, which benefits from the increased end-systolic wall stress [6]. In other words, the slightly increased stiffness appropriately raises wall stress, leading to an increased GWI. This implies myocardial contractility may be enhanced during the early period of the process. However, the pathological processes, including fibroblast proliferation and cardiomyocyte hypertrophy due to chronic afterload elevation, may gradually lead to ventricular hypertrophy, remodeling and eventually LV failure [18–20].

### Global myocardial work in LV remodeling

The abnormality of LV geometry is usually accompanied with increased MWW and lower MWE [21]. GWI and GCW have distinctive values in the evaluation of myocardial function [22]. Hypertensive patients with hypertrophy have lower GLS, GWE and higher GWI and GWW or GCW, implying that the myocardial work-related parameters may reflect the severity of LV remodeling [3, 23]. In the study by Tadic et al. [23], despite the absence of significant differences among the groups, the investigators demonstrated an increasing trend in GWW and a reductive trend in GWE, confirming that the patients were likely at initial etiological (non-pathological) stages. In our study, GWE was preserved due to the appropriate elevation of LV wall stress in the early stages. As LV remodeling proceeded into later stages, decreased GLS and increased GWW led to a poor GWE, which was likely related to elevated ventricular stiffness caused by myocardial impairment.

### Contribution of segmental myocardial work to global myocardial work

During the deterioration of HT, apical myocardial work increases and basal myocardial work decreases gradually in relation to the LV remodeling [18]. Apical myocardial work has also been shown to be increased as a compensation of the impairment of the basal region, especially in hypertensive patients with basal septal hypertrophy [3]. In patients with high blood pressure at peak exercise, the apical myocardial work increased significantly, with an accompanying increase in GCW and GWW, while GWE remained preserved. This implies that the apical myocardial work may contribute to the elevation or preservation of global work [7]. The present study showed that apical MWI and MCW increased in NG and CR groups, with basal or middle segments unchanged. Basal regions had a greater radius of curvature compared to the apical regions, leading to an increased exposure to wall stress as posited by the Laplace Law [24]. As a result, basal wall stress increased dramatically in hypertensive patients. The consequential imbalance between elevated wall stress and locally developed force resulted in decreased local deformation [3]. In other words, the basal myocardium was impaired first while apical segments remained unimpaired, as a compensating mechanism intended to maintain normal global deformation or MWE by enhancing contractility. However, this constant imbalance may trigger severe, regional myocardial impairment, especially in the later stages of LV remodeling. Ultimately, this could result in decreased MWI, MCW and increased MWW across all segments, manifested through a reduced MWE.

### Is myocardial work superior to GLS?

In the present study, for GWI and GCW, there were no statistically significant differences between HT subgroups and control subjects. Conversely, GLS demonstrated a gradual decrease, even in the early stages of remodeling. For ethical reasons, all patients’ medication continued uninterrupted. As a result, the blood pressure was not elevated severely in the cohort enrolled. Previous study has revealed that GWI is increased significantly only in situations where blood pressure > 160 mm Hg [6]. The ROC analysis showed that LS and MWE have a high sensitivity and specificity for predicting the occurrence of hypertensive hypertrophy, and GLS still demonstrated a superior performance. These results further suggested global myocardial work may be not superior to GLS in the cases of normal or slightly elevated afterload.

Nonetheless, segmental myocardial work may potentially be useful in the assessment of myocardial deformation. Particularly, apical and basal myocardial work offer quantitative insights into myocardial function and provide more information about the distribution of myocardial impairment caused by HT.

### Limitations

Some limitations of the current study should be noted: 1) this was a single-center study with small sample size, especially in the CR group. Thus, a larger multi-center study is needed in the future to validate the current findings. 2) For ethical purposes, all patients were enrolled without an interrupted medication course, which resulted in the absence of subjects with a severe elevated blood pressure. Our study was thus only able to describe myocardial work across patients with various patterns of LV geometry with present afterload conditions.

## Conclusion

In the early stages of LV remodeling, apical myocardial work is increased as a compensatory mechanism, playing an important role in the maintenance of global myocardial work. During the later stages, apical myocardial work decreases gradually, resulting an obvious decreased GWE. Myocardial work parameters are useful as non-invasive tools for the assessment of myocardial function. Further, segmental myocardial work analysis offers the opportunity to explore the distribution of myocardial impairment in HT more dynamically.

## Supplementary Information

**Additional file 1. **

## Data Availability

All data used and analyzed in the study are included in this published article (and its Supplementary information files).

## Abbreviations

2D-STE: 2 Dimensional speckle tracking echocardiography
LV: Left ventricular
GWI: Global myocardial work index
GLS: Global longitudinal strain
HT: Hypertension
PSL: Pressure-strain loop
NC: Normal controls
CAD: Coronary artery disease
RWT: Relative wall thickness
LVMI: Left ventricular mass index
NG: Normal geometry
CR: Concentric remodeling
CH: Concentric hypertrophy
EH: Eccentric hypertrophy
LVIDd: Left ventricular internal dimension in diastole
LVIDs: Left ventricular internal dimension in systole
IVST: Interventricular septal thickness
LVPWT: Left ventricular posterior wall thickness
LVEDV: Left ventricular end diastole volume
LVESV: Left ventricular end systolic volume
LAV: Left atrial volume
LVEF: Left ventricular ejection fraction
BSA: Body surface area
ROI: Region of interest
LS: Longitudinal strain
GCW: Global myocardial constructive work
GWW: Global myocardial wasted work
GWE: Global myocardial work efficiency
MWI: Myocardial work index
MCW: Myocardial constructive work
MWW: Myocardial wasted work
MWE: Myocardial work efficiency;
Bas-LS, Mid-LS, Api-LS: Longitudinal strain of basal segments, middle segments, apical segments
Bas-MWI, Mid-MWI, Api-MWI: Myocardial work index of basal segments, middle segments, apical segments
Bas-MCW, Mid-MCW, Api-MCW: Myocardial constructive work of basal segments, middle segments, apical segments
Bas-MWW, Mid-MWW, Api-MWW: Myocardial wasted work of basal segments, middle segments, apical segments
Bas-MWE, Mid-MWE, Api-MWE: Myocardial work efficiency of basal segments, middle segments, and apical segments
OR: Odds ratio
SD: Standard deviation
ANOVA: Analysis of variance
ROC: Receiver operating characteristic
ICC: Interclass correlation coefficient
BSA: Body surface area
ACE-I: Angiotensin-converting enzyme inhibitor
ARB: Angiotensin receptor blocker
CCB: Calcium channel blocker
SBP: Systolic blood pressure
DBP: Diastolic blood pressure
AUC: Area under the curve
CI: Confidence interval
